# Retraction of: Lethal Disseminated Mucorales Infection With Positive Blood Cultures With Purpura Fulminans Complicating Hemophagocytic Lymphohistiocytosis After Chimeric Antigen Receptor T-Cell Therapy

**DOI:** 10.1093/ofid/ofae730

**Published:** 2024-12-17

This is a retraction of: Takahiro Matsuo, Sebastian Wurster, Doina Ivan, Rachel Hicklen, Kelly McConn, Kelli A Bagwell, Fareed Khawaja, Roy F Chemaly, Dimitrios P Kontoyiannis, Lethal Disseminated Mucorales Infection With Positive Blood Cultures With Purpura Fulminans Complicating Hemophagocytic Lymphohistiocytosis After Chimeric Antigen Receptor T-Cell Therapy, *Open Forum Infectious Diseases*, Volume 11, Issue 11, November 2024, ofae647, https://doi.org/10.1093/ofid/ofae647

The authors are retracting this case report because it featured an active clinical trial participant and interpretation of clinical outcomes in that participant that were not validated and/or approved by the principal investigator and the study team. The presented data both was incomplete and certain elements lacked accuracy and therefore led to some conclusions that were inconsistent with those made by the principal investigator and study team. Furthermore, there are contractual and publication obligations in place between the site study team and the corporate entity that supports the multicenter study which the authors regret they have not followed.

